# Resistance of SARS-CoV-2 variants to neutralization by convalescent plasma from early COVID-19 outbreak in Singapore

**DOI:** 10.1038/s41541-021-00389-2

**Published:** 2021-10-25

**Authors:** Bei Wang, Yun Shan Goh, Tessa Prince, Eve Zi Xian Ngoh, Siti Nazihah Mohd Salleh, Pei Xiang Hor, Chiew Yee Loh, Siew Wai Fong, Catherine Hartley, Seow-Yen Tan, Barnaby Edward Young, Yee-Sin Leo, David C. Lye, Sebastian Maurer-Stroh, Lisa F. P. Ng, Julian A. Hiscox, Laurent Renia, Cheng-I Wang

**Affiliations:** 1 Singapore Immunology Network, Agency for Science, Technology and Research (A*STAR), Singapore, Singapore; 2 A*STAR Infectious Diseases Labs (A*STAR ID Labs), Agency for Science, Technology and Research (A*STAR), Singapore, Singapore; 3 Institute of Infection, Veterinary and Ecological Sciences, University of Liverpool, Liverpool, UK; 4 NIHR Health Protection Research Unit in Emerging and Zoonotic Infections, Liverpool, UK; 5 Department of Infectious Diseases, Changi General Hospital, Singapore, Singapore; 6 National Centre for Infectious Diseases, Singapore, Singapore; 7 Department of Infectious Diseases, Tan Tock Seng Hospital, Singapore, Singapore; 8 Lee Kong Chian School of Medicine, Nanyang Technological University, Singapore, Singapore; 9 Yong Loo Lin School of Medicine, National University of Singapore and National University Health System, Singapore, Singapore; 10 Bioinformatics Institute, Agency for Science Technology and Research (A*STAR), Singapore, Singapore; 11 Department of Biological Sciences, National University of Singapore, Singapore, Singapore; 12 Department of Biochemistry, Yong Loo Lin School of Medicine, National University of Singapore, Singapore, Singapore; 13 School of Biological Sciences, Nanyang Technological University, Singapore, Singapore

**Keywords:** SARS-CoV-2, Antibodies

## Abstract

The rapid spreading of SARS-CoV-2 variants B.1.1.7 originated from the United Kingdom and B.1.351 from South Africa has contributed to the second wave of COVID-19 cases in the respective countries and also around the world. In this study, we employed advanced biochemical and virological methodologies to evaluate the impact of Spike mutations of these strains on the degree of protection afforded by humoral immune responses following natural infection of the ancestral SARS-CoV-2 strain during the early stages of the outbreak. We found that antibody-mediated neutralization activity was partially reduced for B.1.1.7 variant and significantly attenuated for the B.1.351 strain. We also found that mutations outside the receptor-binding domain (RBD) can strongly influence antibody binding and neutralization, cautioning the use of solely RBD mutations in evaluating vaccine efficacy. These findings highlight an urgent need to develop new SARS-CoV-2 vaccines that are not based exclusively on the ancestral SARS-CoV-2 Spike gene sequence.

## Introduction

Coronavirus disease 2019 (COVID-19), caused by the severe acute respiratory syndrome coronavirus 2 (SARS-CoV-2), has posed a major public health threat since its outbreak in Wuhan in January 2020^1^. Different SARS-CoV-2 variants, such as B.1.1.7 and B.1.351, have since emerged as a result of natural evolution and immune escape^2^. The B.1.1.7 strain was first isolated in England in September 2020 and soon became the dominant strain in the UK and hence was referred to as United Kingdom COVID-19 variant (UK strain)^3,4^. The B.1.351 strain, also known as the South African COVID-19 variant (SA strain), was first detected in South Africa in October 2020 and rapidly drove the second wave of COVID-19 pandemic in the country^5^.

The UK and SA variants share two common mutations, the D614G mutation and an N501Y mutation in the receptor-binding domain (RBD) of the Spike protein and hence are also referred to as 501Y.V1 and 501Y.V2, respectively^6^. Besides the N501Y mutation, the UK strain Spike protein also contains two distinct deletions at the N-terminal domain (NTD) (69–70del and 144–145del) and a panel of single amino acid changes, including the A570D, D614G, P681H, T716I, S982A, and D1118H mutations^5,7^. On the other hand, the Spike protein of the SA strain contains changes of the 242–245del, the D80A, and R246I substitutions in NTD, two more point mutations within RBD—K417N and E484K, and one mutation A701V near the furin cleavage site. Both the UK and SA strains have been reported to display enhanced transmissibility, partially because the variants attach more easily to cells expressing human ACE2^3,8–11^.

There are growing concerns that these variants may have a negative impact on the efficacy of the global vaccine programs because all current vaccines used the Spike sequences of the ancestral Wuhan strain^12–17^. In fact, neutralization efficacy against these emerging variants either from human convalescent sera or from human vaccinated sera, isolated from European and American cohort, have been reported to be significantly diminished^6,7,18–24^. Sporadic reports have also been shown to be associated with Middle East countries^25,26^. However, whether the wild-type (WT) SARS-CoV-2 infection in a southeast Asian cohort can provide protection against these newly emerged variants remains largely unknown. In addition, for antibodies currently in therapeutic development, there have been contrasting results with some antibodies being affected by mutations and others not^7,19^. Therefore, in this study, we aim to evaluate the binding and neutralizing potency of the plasma samples from convalescent COVID-19 patients infected with the WT SARS-CoV-2 in Singapore collected between January and April in 2020^27^ against the UK and SA variants. We also studied the contribution of RBD-only mutations (K417N/E484K/N501Y triple mutant) of the SA strain, as well as a few single or double point mutations in these Spike variants to the loss of antibody activities. These findings provide initial insight on the degree of protection against these variants that vaccines based on the WT SARS-CoV-2 might elicit in a southeast Asian cohort, such as Singapore.

## Results

### Profiles of antibody binding against full-length variant S proteins

**Fig. 1:**
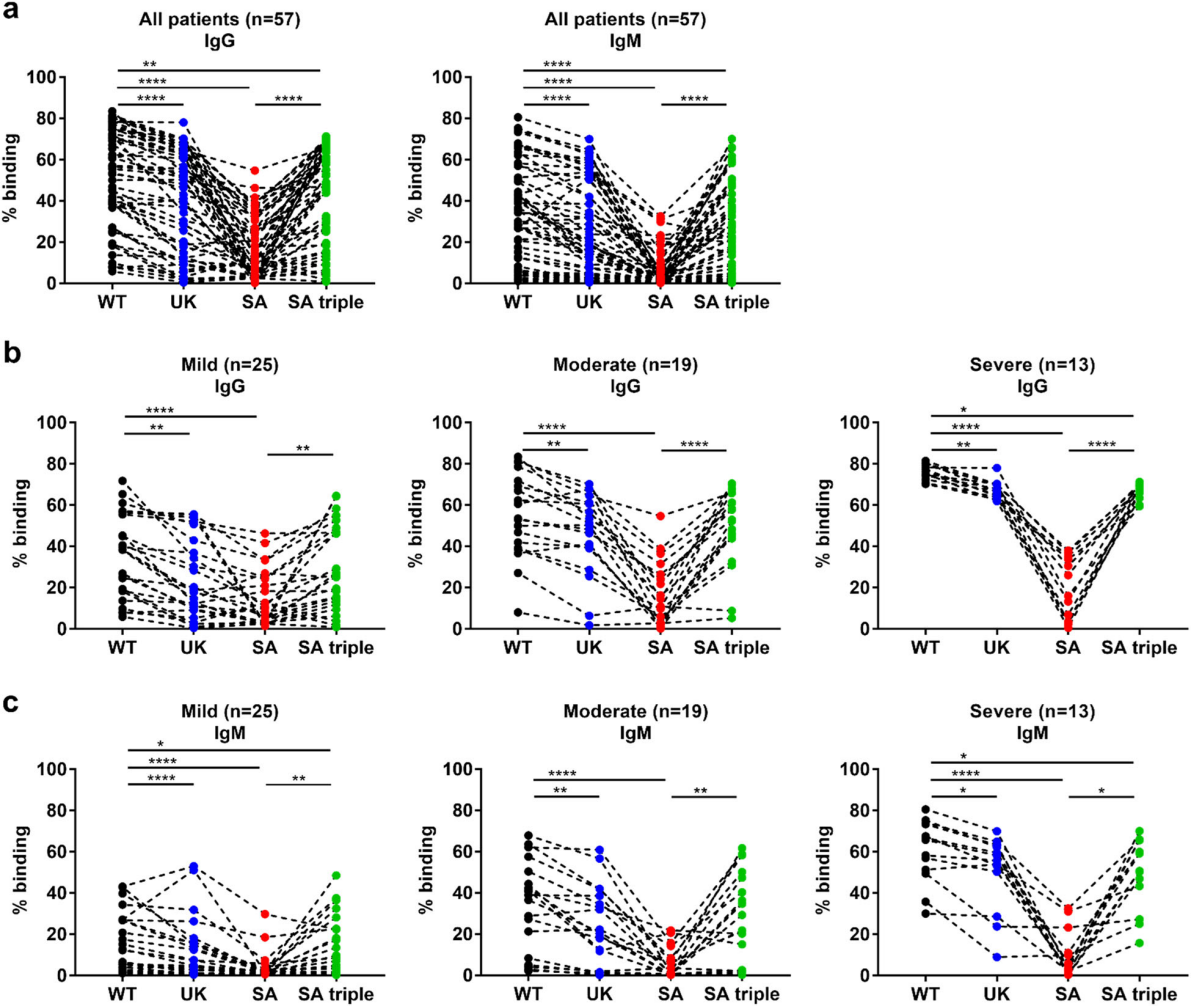
S protein-specific antibody profile against WT, UK, SA and RBD-specific SA triple variants. Plasma samples were collected from COVID-19 patients with SARS-CoV-2 infections (*n* = 57) at median 31 days post-illness onset (pio) (mild, *n* = 25; moderate, *n* = 19; severe, *n* = 13). **a** Samples were screened at 1:100 dilution for specific IgG and IgM against the different variants of full-length SARS-CoV-2 S protein expressed on the surface of HEK293T cells. IgG (**b**) and IgM (**c**) binding across the three disease severity groups. Data were shown as mean ± SD of two independent experiments. Statistical analysis was carried out on the paired samples using the Friedman test, followed by post hoc Dunn’s multiple comparison tests. *P* values for comparisons between the groups are shown, where * indicates *P* ≤ 0.05, ** indicates *P* ≤ 0.01, *** indicates *P* ≤ 0.001, and **** indicates *P* ≤ 0.0001.

To characterize the antibody activity profile of COVID-19 patients, we previously reported a flow cytometry-based assay to detect antibodies against S protein (SFB assay), using lentivirus transduced cells expressing the full-length WT S protein on the cell surface^28^. In order to examine if the humoral response of patients recovered from a prior SARS-CoV-2 infection is specific for the WT strain or is able to detect other strains, we applied the SFB assay to detect antibodies against S protein of other SARS-CoV-2 variants. These include B.1.1.7 bearing changes of the 69–70 deletions, 144–145 deletions, as well as N501Y, A570D, D614G, P681H, T716I, S982A, and D1118H substitutions (UK); B.1.351 carrying signature changes of 242–245 deletions, D80A, R246I, K417N, E484K, N501Y, D614G, and A701V substitutions (SA); and a triple mutant carrying the RBD-specific mutations K417N, E484K, and N501Y (SA triple). Using the cell lines that express these S protein variants, we determined the antibody response of 57 convalescent individuals with a median of 31 days post-illness onset (pio), corresponding to the peak of antibody response^27,29,30^. These COVID-19 patients, mostly suffering from a WT SARS-CoV-2 infection^27^, had stronger S protein-binding IgG antibodies against the WT strain than to the UK and SA variants (Fig. 1a). The decrease in IgG and IgM binding was most prominent with the SA variant. Interestingly, when we examined the level of S protein-specific antibody binding to RBD-specific SA triple variant, the decrease in IgG and IgM binding was markedly smaller than to the full-mutation SA variant. We then went to stratify the patients by their clinical severity during hospitalization: mild (no pneumonia on chest radiographs [CXR], *n* = 25), moderate (pneumonia on CXR without hypoxia, *n* = 19), and severe (pneumonia on CXR with hypoxia [blood oxygen levels ≤94%], *n* = 13). No correlation between the extent of decrease in IgG or IgM binding and the disease severity was found in any of the variants (UK, SA, and SA triple)—we found a similar trend in both IgG and IgM binding in all three severity groups (Fig. 1b, c).

### Antibody neutralization against variant pseudoviruses

**Fig. 2:**
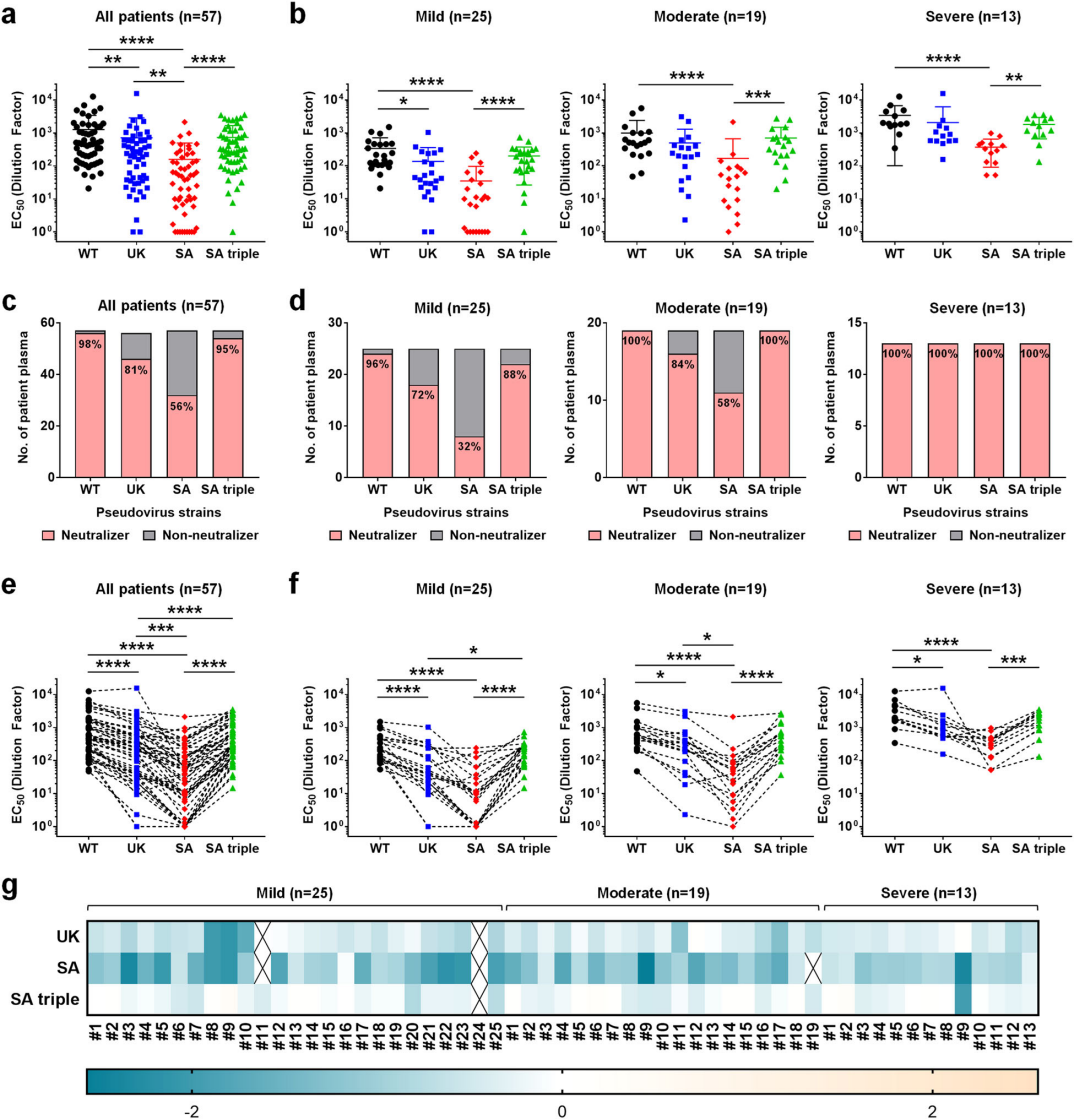
Pseudovirus neutralization profiles of 57 COVID-19 convalescent plasma samples. Anti-SARS-CoV-2 neutralizing antibodies from 57 COVID-19 patients during the SARS-CoV-2 outbreak in Singapore were assessed using pseudovirus expressing SARS-CoV-2 Spike glycoprotein of either the original Wuhan strain (WT), UK strain B.1.1.7 (UK), SA strain B.1.351 (SA), and SA triple mutant K417N/E484K/N501Y (SA triple). All EC_50_ values of neutralization were calculated by variable slope four-parameter nonlinear regression model using Graphpad PRISM 7 Software with top and bottom constraints set at 100 and 0% respectively. **a**, **b** Dot plots of EC_50_ values of neutralization against WT (black circles), UK (blue squares), SA (red diamonds), and SA triple (green triangles) pseudoviruses from all 57 COVID-19 patients (**a**) or presented in three different severity groups of mild (*n* = 25), moderate (*n* = 19), and severe (*n* = 13) (**b**). Statistical analysis was carried out to compare any two different pseudoviruses using Kruskal–Wallis tests followed by post hoc Dunn’s multiple comparisons tests (*P ≤ 0.05, **P ≤ 0.01, ***P ≤ 0.001, ****P ≤ 0.0001). **c**, **d** Each individual patient sample was arbitrarily defined as a “Neutralizer” (pink bar) if the neutralizing EC_50_ is equal to or bigger than 20 (dilution factor) or “Non-neutralizer” (gray bar) if the neutralizing EC_50_ is less than 20 (dilution factor). The numbers indicate the percentage of neutralizers. Distributions of “Neutralizers” and “Non-neutralizers” were presented across all 57 patients (**c**) or in each different disease severity groups (**d**). **e**, **f** Paired analysis of EC_50_ values of neutralization was carried out using Friedman test followed by post hoc Dunn’s multiple comparisons tests (*P ≤ 0.05, ***P ≤ 0.001, ****P ≤ 0.0001). Comparison between WT (black circles), UK (blue squares), SA (red diamonds), and SA triple (green triangles) pseudoviruses from all 57 COVID-19 patients (**e**) or presented in three different severity groups of mild (*n* = 25), moderate (*n* = 19), and severe (*n* = 13) (**f**). **g** Log_10_ values of fold increase (positive value) or fold decrease (negative value) in neutralization EC_50_ of UK, SA, and SA triple pseudoviruses, relative to the WT pseudovirus are presented as heat maps with darker colors implying greater changes. The samples were organized into three different severity groups of mild (*n* = 25), moderate (*n* = 19), and severe (*n* = 13). Yellow, increase; Blue, reduction. An X indicates that the EC_50_ value cannot be determined.

To evaluate the effects of SARS-CoV-2 strain variation on antibody neutralization, we generated pseudoviruses expressing SARS-CoV-2 Spike glycoprotein of either the WT strain, B.1.1.7 strain (UK), or B.1.351 strain (SA) together with the RBD triple mutant (SA triple). The neutralization EC_50_ values (Supplementary Table 1) of the patients against the pseudoviruses of different strains were interpolated from the respective dose-response neutralization titration curves (Supplementary Fig. 1). Notably, although most plasma samples were still able to neutralize variants of SARS-CoV-2 pseudoviruses at the highest concentration (20 times dilution), the potency of all 57 patient samples against both UK and SA strains decreased significantly when compared to the original WT strain (Supplementary Table 1 and Fig. 2a). The most significant decrease occurred against the SA strain. Interestingly, SA triple mutants did not exhibit significant overall changes in neutralization EC_50_ except in a small subset of patients (Fig. 2a, c), suggesting that the SA triple mutant bearing only the RBD mutations is not the best indicator to represent the full SA variant carrying all significant changes. Next, neutralization EC_50_ values of 57 patients were categorized into three groups based on their clinical severity during hospitalization. Consistent with our previous report^27^ and several other studies^31–33^, patients from the severe group showed higher levels of neutralizing antibodies as compared to the mild and moderate patients (Fig. 2b). Hence, even though there is a significant decrease in potency against the SA strain in the severe group, the plasma samples from all 13 patients still retained greater than 50% neutralization at the highest plasma concentration assayed (20 times dilution) (Supplementary Fig. 1c and Fig. 2d). This is in contrast to the mild group, in which as high as 68% of patients (17 out of 25) lost more than 50% of neutralizing activity against SA strain at the same plasma concentration and hence are defined as “Non-neutralizers” (Supplementary Fig. 1c and Fig. 2d). Similar trends were found in the UK strain, albeit less significant than the SA strain in general (Supplementary Fig. 1b and Fig. 2d). Nevertheless, when applying individual paired analysis, we found that the differences between the UK or SA strain and the WT strain were significant across all patients (Fig. 2e) and in all severity groups (Fig. 2f). Again, no significant differences were observed between the SA triple mutant and the WT strain in the paired analysis (Fig. 2e, f). Fold changes in neutralizing EC50 in the log_10_ scale relative to the WT pseudovirus for UK, SA, and SA triple across all 57 patients are also presented by heat map and shown in Fig. 2g. Negative values reflected a decrease in neutralizing potency and were most obvious in the SA group.

### Antibody neutralization against single- or double-point mutants

**Fig. 3:**
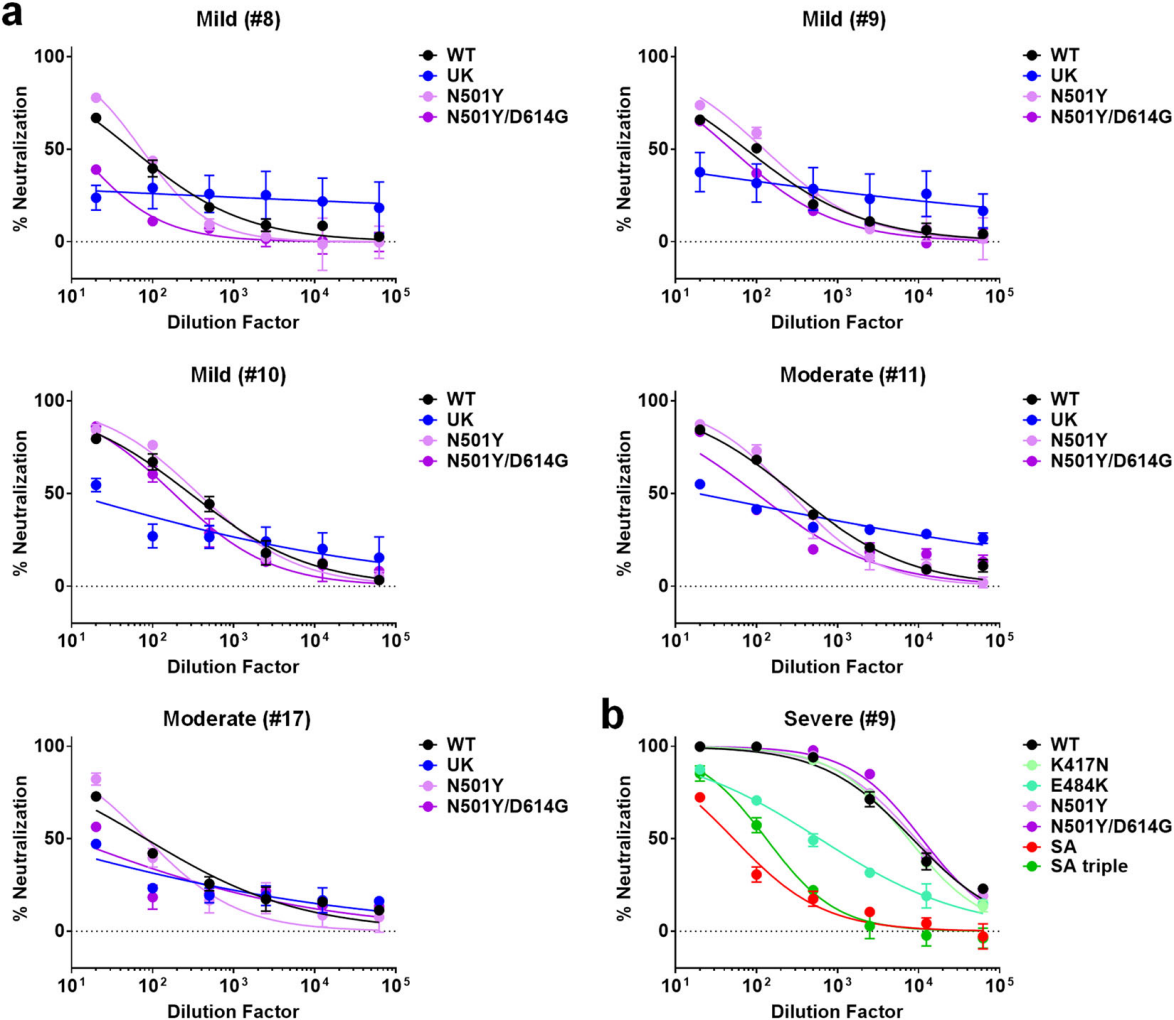
Pseudovirus neutralization profiles of selected convalescent patient plasma against single or double point mutants. **a** Pseudovirus neutralization assays of five patients, including #8, #9, and #10 from the mild group and #11, #17 from the moderate group. Anti-SARS-CoV-2 neutralizing antibodies from the selected COVID-19 patients were assessed using pseudovirus expressing SARS-CoV-2 Spike glycoprotein of either the original WT strain (black), UK strain (blue), N501Y single point mutant (light purple), and N501Y/D614G double mutant (dark purple) at 1:20 to 1:62500 dilutions. **b** Pseudovirus neutralization assays of patient #9 from the severe group. Anti-SARS-CoV-2 neutralizing antibodies from the selected COVID-19 patients were assessed using pseudovirus expressing SARS-CoV-2 Spike glycoprotein of either the original WT strain (black), SA strain (red), SA triple strain (green), K417N single point mutant (lime green), E484K single point mutant (light green), N501Y single point mutant (light purple), and N501Y/D614G double mutant (dark purple) at 1:20 to 1:62500 dilutions. Lines represent nonlinear regression fit and data were shown as mean ± SD of two independent experiments.

We further investigated the effects of mutations in the RBD on the neutralization potency. Patients #8, #9, and #10 from the mild group, and patients #11, #17 from the moderate group exhibited a significant decrease (>10-fold) in neutralization potency against UK strain (Fig. 2g) and were chosen for the study of the effect of the N501Y mutation in the RBD. We found that this single mutation N501Y did not account for the loss of activity against the UK strain, and only partially so in the context of D614G mutation (Fig. 3a). Similarly, the RBD triple mutant K417N/E484K/N501Y did not account for the loss of neutralizing activity of the SA strain across patients of various severity outcomes, with only one prominent exception, patient #9 in the severe group (Fig. 2g), who is sensitive to the E484K mutation (Fig. 3b).

### Antibody neutralization against live viruses

**Fig. 4:**
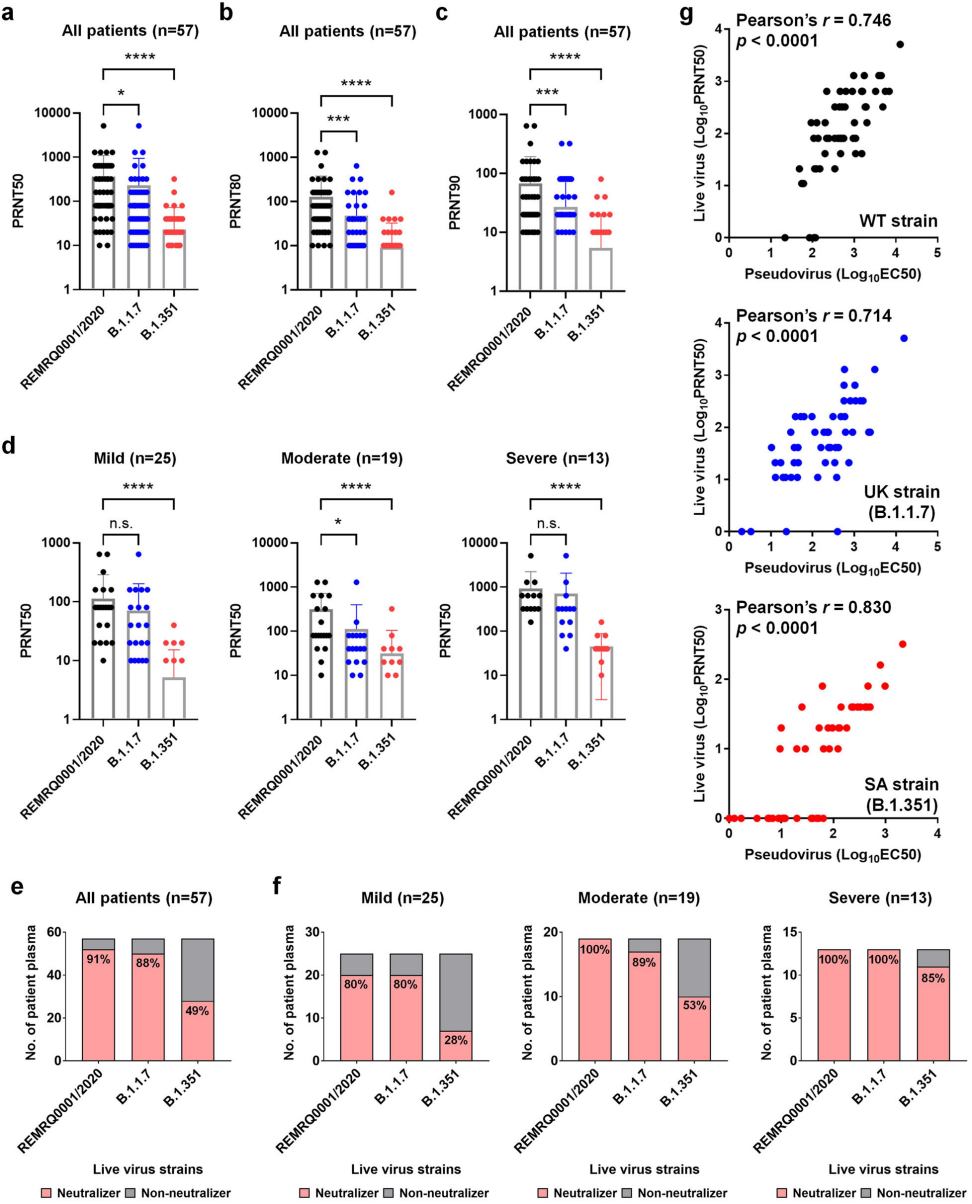
Live virus neutralization profiles of 57 COVID-19 convalescent plasma samples. The neutralization activity of plasma samples against three different variants of SARS-CoV-2, a B lineage virus (REMRQ0001/2020, expressing WT Spike protein), a B.1.1.7 lineage virus, and a B.1.351 lineage virus. Each dot represents an individual plasma sample. For plasma samples that did not neutralize the virus, a value of “0” was assigned and hence are not plotted in the figures on a logarithmic scale. **a** The PRNT50 titers of COVID-19 patients (*n* = 57). **b** The PRNT80 titers of COVID-19 patients (*n* = 57). **c** The PRNT90 titers of COVID-19 patients (*n* = 57). **d** The PRNT50 titers of 57 COVID-19 patients presented in three different severity groups of mild (*n* = 25), moderate (*n* = 19), and severe (*n* = 13). Statistical analysis was carried out to compare any two different live viruses using Kruskal–Wallis tests followed by post hoc Dunn’s multiple comparisons tests (*P ≤ 0.05, ***P ≤ 0.001, ****P ≤ 0.0001, n.s. not significant). Each individual patient sample was arbitrarily defined as a “Neutralizer“ (pink bar) if the live virus-neutralizing PRNT50 is equal to or bigger than 10 (dilution factor) or “Non-neutralizer“ (gray bar) if the neutralizing PRNT50 was set at “0”. The numbers indicate the percentage of neutralizers. Distributions of “Neutralizer” and “Non-neutralizers” were presented across all 57 patients (**e**) or in each different disease severity groups (**f**). **g** Correlation analysis of Log_10_EC50 values of pseudovirus neutralization assay and Log_10_PRNT50 values of live virus neutralization assay against WT strain (top panel), UK strain (B.1.1.7, middle panel), and SA strain (B.1.351, bottom panel). For log transformation, a common rule of log(1 + x) expansion was applied. The Pearson’s correlation coefficient (Pearson’s *r*) and *p* values were calculated using PRISM.

Lastly, we proceeded to examine the neutralizing activity of the 57 convalescent patient plasma samples against WT, B.1.1.7, and B.1.351 live virus isolates. The results corroborated the pseudovirus assay data, with dampened neutralizing potency against both B.1.1.7 strain and B.1.351 strain, compared to a strain isolated from Liverpool (REMRQ0001/2020), bearing the same Spike protein sequence as the ancestral Wuhan strain. The more prominent decrease in PRNT50, PRNT80, and PRNT90 values was also observed with the B.1.351 authentic virus (Fig. 4a–c). Again, when the patients were grouped according to their disease severity, we observed a consistent trend of increased PRNT50 values in moderate to severe diseases (Fig. 4d). This was also reflected by the increase in the numbers of “Neutralizers” in more severe patients across all three different virus strains (Fig. 4e, f). Finally, we performed a correlation analysis by comparing the logarithmic values of EC50s derived from the pseudovirus neutralization assay (Supplementary Table 1) and the PRNT50s of the live virus neutralization assay (Supplementary Table 2), which revealed a strong positive correlation of the two assays with the Pearson’s correlation coefficient (Pearson’s *r*) ranging from 0.746 against the WT strain, 0.714 against the B.1.1.7 UK strain, to a high number at 0.830 against the B.1.351 SA strain (Fig. 4g).

## Discussion

The development and rapid dissemination of SARS-CoV-2 viral variants such as those originated from the United Kingdom and South Africa has generated significant concerns globally on the degree of protection afforded by humoral immune responses elicited by natural infection or vaccination. There have been a number of studies that have demonstrated a significant decrease in antibody responses against B.1.1.7 UK strain and B.1.351 SA strain compared with the ancestral and D614G variant strains, with B.1.351 SA strain showing the greatest decrease in neutralization potency^6,7,19,20^. Using a Singapore cohort of 57 patients, we have previously found that the impact of the D614G mutation is very limited and concluded that neutralizing antibodies elicited by the WT SARS-CoV-2 infection provided strong cross-protection against the D614G variant^27^, while other studies reported a moderate increase of antibody neutralization potency in COVID-19 convalescent patient sera against the D614G variant^34,35^. It is still unclear if the convalescent patients from South East Asia also have a similar immune profile as convalescent patients from the other continents, displaying antibody resistance to the UK and SA strains, and more importantly, if the WT viral infection during the first wave of COVID-19 outbreak in Singapore could provide protection against these newly emerged variants.

In this study, we constructed Spike mutations of the B.1.1.7 UK strain and B.1.351 SA strain into the backbone of the ancestral Wuhan strain. Using the S protein-based flow cytometry^28^ and pseudovirus neutralization assays^27^, we concluded that, similar to the American and European cohort studies, mutations in the Spike protein greatly impact the binding and neutralization capabilities of antibodies from the convalescent patients in the early outbreak in Singapore (Figs. 1, 2, 4)^27^. All convalescent patient samples exhibited a partial reduction in antibody responses against the B.1.1.7 UK variant and a more prominent reduction against the B.1.351 SA strain. The binding efficiency profiles largely mirrored the neutralization potency profiles in all severity groups (Figs. 1, 2, 4), and the lentiviral vector-based pseudovirus assay correlated well with the live virus-based plaque reduction assay (Fig. 4g)^36,37^.

Consistent with our previous study^27^ as well as results from recent reports^31–33,38^, we found that the neutralization titers are generally higher in patients suffering from severe symptoms than those with only mild conditions. Even with a significant decline in neutralization potency, all 13 patients with severe symptoms have antibody titers high enough to achieve more than 50% neutralization at the highest serum concentration (20 times dilution) in the pseudovirus neutralization assay (Fig. 2d). While there is a clear correlation between neutralizing antibody titers and the disease severity, the cause–effect relationship remains elusive. Some hypothesized that uncontrolled viral replication led to hyper-inflammation with high levels of pro-inflammatory cytokine secretion resulting in overproduction of antibodies^31,39^. On the other hand, it was recently shown that patients with severe diseases had anti-SARS-CoV-2 antibodies that function to prevent the induction of interferon-stimulated genes and thus play the immune system against itself^40^. In a previous study, however, patients with severe SARS symptoms were found to accumulate predominately pro-inflammatory macrophages in the lungs. Their sera, often containing high levels of neutralizing antibodies, enhanced virus-induced IL-8 and MCP1 production by human monocyte-derived macrophages that were polarized toward a wound-healing phenotype, whereas blockade of FcγR reduced these pro-inflammatory effects, suggesting pathological roles played by the antibodies^41^. While SARS and COVID-19 display distinct clinical presentations, it’s reasonable to suspect that they may share similar antibody-mediated pathological mechanisms.

Using an RBD-specific K417N/E484K/N501Y triple mutant (SA triple), we found no significant difference between the SA triple mutant and WT strain in all three different severity groups of patients, unlike the B.1.351 SA strain. The only exception is patient #9 in the severe group, which is significantly affected by the E484K mutation. It is also interesting to note that the addition of D614G mutation to the N501Y single mutant strain only slightly affected the neutralization potency and was not sufficient to account for the activity loss to the UK strain. In addition, there is no significant difference in the neutralization potency against all pseudovirus or live virus strains between D614 and G614 virus infection status (Supplementary Fig. 2), which further affirms our previous finding^27^. Our results indicated that mutations beyond the RBD in the B.1.351 SA strain and the B.1.1.7 UK strain may induce long-range allosteric structural changes that influence antibody binding and neutralization. For example, recent structural studies revealed that substitution of the D614 with glycine (the D614G variant) enhanced Spike trimer stability^42^ and allowed more RBDs to assume open orientation to facilitate ACE2 engagement^43^, explaining its higher infectivity and transmissibility^44^. Alternatively, mutations outside the RBD may abolish the neutralizing ability of the antibodies raised against the WT SARS-CoV-2 that recognize the epitopes near or at the sites of mutation. For example, neutralizing antibodies binding to the NTD of the Spike protein, such as FC05 with the binding epitope located near the R246I and del244-245 changes (Supplementary Fig. 3)^45^, may become inactive against the South African strain B.1.351. Furthermore, disruption of antibody binding at certain positions in S2 may prevent them from blocking the folded helixes from undergoing drastic conformation changes into the extended helix, a structural transition required to expose the fusion peptide in the post-fusion conformation^46^.

Taken together, our findings suggested that the previous conclusions drawn from the use of the viral variants containing only RBD mutations to evaluate the efficacy of the vaccines against the UK or SA strain may be misleading^47,48^. These complex findings highlight not only the structural plasticity of the viral Spike proteins but also the variations of immune responses elicited by patients. In addition, while governments around the world are speeding up to have COVID-19 vaccines rolled out efficiently to the majority of the population, it is of great concern that all currently approved vaccines and those still in development are based on the ancestral Spike sequence^14^. Our findings has shed light on the potential reduced vaccine efficacy of current COVID-19 vaccines against the emerging viral variants and highlighted the urgent need to develop new COVID-19 vaccines that are not exclusively based on the ancestral Spike sequence.

## Methods

### Ethics statement

The design and protocols of this study for convalescent COVID-19 patient cohorts have been approved by the National Healthcare Group (NHG) Domain Specific Review Board (DSRB) under study number IRB#2012/00917 and performed following the ethical guidelines. Written informed consent was obtained from participants in accordance with the tenets of the Declaration of Helsinki. All samples received were collected under the Singapore Infectious Diseases Act, which allows epidemiological studies and the use of data for analysis to control disease outbreaks.

### Plasma samples for COVID-19 patients

A total of 57 patients who tested PCR-positive for SARS-CoV-2 in nasopharyngeal swabs in Singapore were recruited into the study from January to March 2020^27^ during the initial phase of the COVID-19 pandemic (before any COVID-19 vaccines are approved and available). Patients were categorized into three groups based on clinical severity during hospitalization: mild (no pneumonia on chest radiographs [CXR], *n* = 25), moderate (pneumonia on CXR without hypoxia, *n* = 19), and severe (pneumonia on CXR with hypoxia [desaturation to ≤94%], *n* = 13). The whole blood of patients was collected in BD Vacutainer CPT^TM^ tubes (BD Biosciences, Franklin Lakes, NJ, USA) and centrifuged at 1700×*g* for 20 min to obtain plasma fractions. Plasma samples were heat-inactivated at 56 °C for 30 min for virus inactivation.

### Generation of constructs expressing Spike genes of different variants

The pTT5LnX-CoV-SP (expressing SARS-CoV-2 Spike protein, Genbank: YP_009724390.1, a kind gift from Dr. Brendon John Hanson, DSO National Laboratories) was used as a template to generate Spike genes of different variants using QuickChange Lightning Multi Site-Directed Mutagenesis Kit (Agilent, Cat#210513). The primers were listed in Supplementary Table 3.

### Generation of S protein-expressing cell line

HEK293T cells expressing WT SARS-CoV-2 S protein were generated as previously described^28^. For HEK293T cell expressing the UK, SA, or RBD-specific SA triple mutation variants of SARS-CoV-2 S protein, the three variants of the full-length S gene were each cloned into pHIV-eGFP transfer plasmid, via the XbaI and BamHI sites, upstream of an IRES (internal ribosome entry site) and an eGFP gene (Supplementary Table 3). The transfer plasmid, pHIV-SARS-CoV-2-(UK)SP-eGFP, pHIV-SARS-CoV-2-(SA)SP-eGFP, or pHIV-SARS-CoV-2-(SA triple)SP-eGFP, was then co-transfected with the packaging and envelope plasmids pMD2.G (a gift from Didier Trono, Addgene #12259), pMDLg/pRRE (a gift from Didier Trono, Addgene #12251), and pRSV-Rev (a gift from Didier Trono, Addgene #12253) into HEK293T cells (Cat#CRL-3216) using EndoFectin Lenti (GeneCopoeia, Cat#EF001). After 8 h, the medium (DMEM + 10% FBS) was changed and the lentiviral particles in the supernatant were collected following a further 48 h incubation. Cells were transduced by adding the lentiviral supernatant and 8 μg/ml polybrene (Sigma-Aldrich, Cat#H9268), then centrifuging at 1200x*g* for 1 h at room temperature. After a further 48 h incubation, eGFP-expressing HEK293T cells were sorted, expanded, and cryopreserved.

### S protein flow cytometry-based assay (SFB assay) for antibody detection

The SFB assay was performed as previously described^28^. Briefly, S protein-expressing cells were seeded at 1.5 × 10^5^ cells per well in 96-well V-bottom plates (Thermo Fisher Scientific, Cat#249570). After incubating the cells with diluted human plasma (1:100 in 10% FBS), the cells were incubated with a double stain, consisting of Alexa Fluor 647-conjugated anti-human IgM (Thermo Fisher Scientific, Cat#A21249; diluted 1:500), or IgG (Thermo Fisher Scientific, Cat#A21445; diluted 1:500) and propidium iodide (PI, Sigma-Aldrich, Cat#P4170; diluted 1:2500). Cells were read on BD Biosciences LSR4 laser and analyzed using FlowJo (Tree Star). Cells were gated on: (1) FSC-A/SSC-A to exclude cell debris, (2) FSC-A/FSC-H to select for single cells, (3) FSC-A/PI to select for live cells (PI-negative population), (4) FITC/Alexa Fluor 647. Binding is determined by the percentage of GFP-positive S protein-expressing cells that are bound by specific antibody, indicated by the events that are Alexa Fluor 647- and FITC-positive. A figure exemplifying the gating strategy is provided (Supplementary Fig. 4). Quantification of binding of specific antibody binding to cells were analysed following our previous study^28^.

### SARS-CoV-2 pseudovirus production

The lentiviral-based pseudovirus were produced as previously described in refs. ^27,36^. Briefly, using the third-generation lentivirus system, pseudotyped viral particles expressing SARS-CoV-2 S proteins of WT strain or other variants were generated by reverse transfection of 3 × 10^7^ of HEK293T cells (Cat#CRL-3216) with 12 µg pMDLg/PRRE (a gift from Didier Trono, Addgene #12251), 6 µg pRSV-Rev (a gift from Didier Trono, Addgene #12253), 24 μg pHIV-Luc-ZsGreen (a gift from Bryan Welm, Addgene #39196), and 12 µg pTT5LnX-CoV-SP using Lipofectamine 2000 transfection (Invitrogen, Cat#11668-019). Three days later, the viral supernatant was harvested by centrifugation to remove cell debris and filtered through a 0.45 µm filter unit (Sartorius), and the viral titers were quantified with Lenti-X^TM^ p24 Rapid Titers Kit (Takara Bio, Cat #632500).

### Pseudovirus neutralization assay

The pseudovirus neutralization assay was performed as previously described with slight modifications^27^. CHO-ACE2 cells (a kind gift from Professor Yee-Joo Tan, Department of Microbiology, NUS & IMCB, A*STAR, Singapore)^49^ were seeded at 1.8 × 10^4^ per well in a 96-well black polystyrene TC-treated microplate (Corning, Cat#3904) in culture medium without Geneticin. After overnight culture, serially diluted heat-inactivated plasma samples (20-fold dilutions from 1:20 to 1:62500) were incubated with equal volume of pseudovirus expressing SARS-CoV-2 S proteins of either original WT or variant strain (12 ng of p24) at a final volume of 50 µl at 37 ˚C for 1 h, before being added to pre-seeded CHO-ACE2 cells in duplicate. After 1 h of pseudovirus infection at 37 °C, cells were topped up with 150 µl of culture media and cultured for additional two days. Cells were washed with PBS, lysed with 1x Passive Lysis Buffer (Promega, Cat#E1941) with gentle shaking at 125 rpm for 30 min at 37 ˚C and the luciferase activity was assessed with Luciferase Assay System (Promega, Cat#E1510) on a GloMax Luminometer (Promega). The percent neutralization was calculated by normalizing the raw RLU values to the average value of RLU in virus only control wells.

### Live virus inhibition assay

Viral stocks of the SARS-CoV-2/human/Liverpool/REMRQ0001/2020 isolate (Genbank ID MW041156.1), the B.1.1.7 isolate, and the B.1.351 isolate were generated in Vero/hSLAM cells with Dulbecco’s minimal essential medium (DMEM) (Sigma) containing 4% fetal bovine serum (FBS) (Sigma), 0.05 mg/ml gentamicin (Merck), and 0.4 mg/ml geneticin (G418; Thermo Fisher) and harvested 72 h postinoculation. Virus stocks were aliquoted and stored at −80 °C as previously described^50^. PRNTs were performed using African green monkey kidney C1008 (Vero E6) cells (Public Health England, PHE). Sera were heat-inactivated at 56 °C for 1 h and stored at −20 °C until use. DMEM containing 2% FBS and 0.05 mg/mL gentamicin was used for serial twofold dilutions of patient plasma samples. SARS-CoV-2 at 800 PFU/mL was added to an equal volume of diluted plasma and incubated at 37 °C for 1 h. The virus-plasma dilution was inoculated onto Vero E6 cells in duplicate and incubated at 37 °C for 1 h. They were then overlaid with agarose as in standard plaque assays. Cells were incubated for 72 h at 37 °C and 5% CO_2_ before being fixed with 10% formalin and stained with crystal violet solution (Sigma-Aldrich). PRNT_90/80/50_ was determined by the highest dilution with a 90/80/50% reduction in plaques compared to the control.

### Statistical analysis

Statistical analysis was done using GraphPad Prism version 7.03 (GraphPad Software). To compare between multiple groups, Kruskal–Wallis tests and post hoc tests using Dunn’s multiple comparison tests were used to identify significant differences. For paired analysis between different variants, Wilcoxon matched-pairs signed-rank tests were used. *P* values less than 0.05 are considered significant.

### Reporting Summary

Further information on research design is available in the Nature Research Reporting Summary linked to this article.

## Supplementary information

Supplementary Information

Reporting Summary

## Data Availability

The data generated and analyzed during the current study are available from the corresponding author upon reasonable request.

## Materials availability

All requests for biological materials and other reagents should be directed to and will be fulfilled by the corresponding authors (Cheng-I Wang or Laurent Renia) with a completed Materials Transfer Agreement.
