# Supplementary material for: Resistance of SARS-CoV-2 variants to neutralization by convalescent plasma from early COVID-19 outbreak in Singapore
Source: NPJ Vaccines. 2021 Oct 25;6:125. doi: 10.1038/s41541-021-00389-2 (PMC8546091; doi:10.1038/s41541-021-00389-2)
Supplement: Supplementary file 1 — Supplementary Information [file 41541_2021_389_MOESM1_ESM.pdf]

SUPPLEMENTARY FIGURES

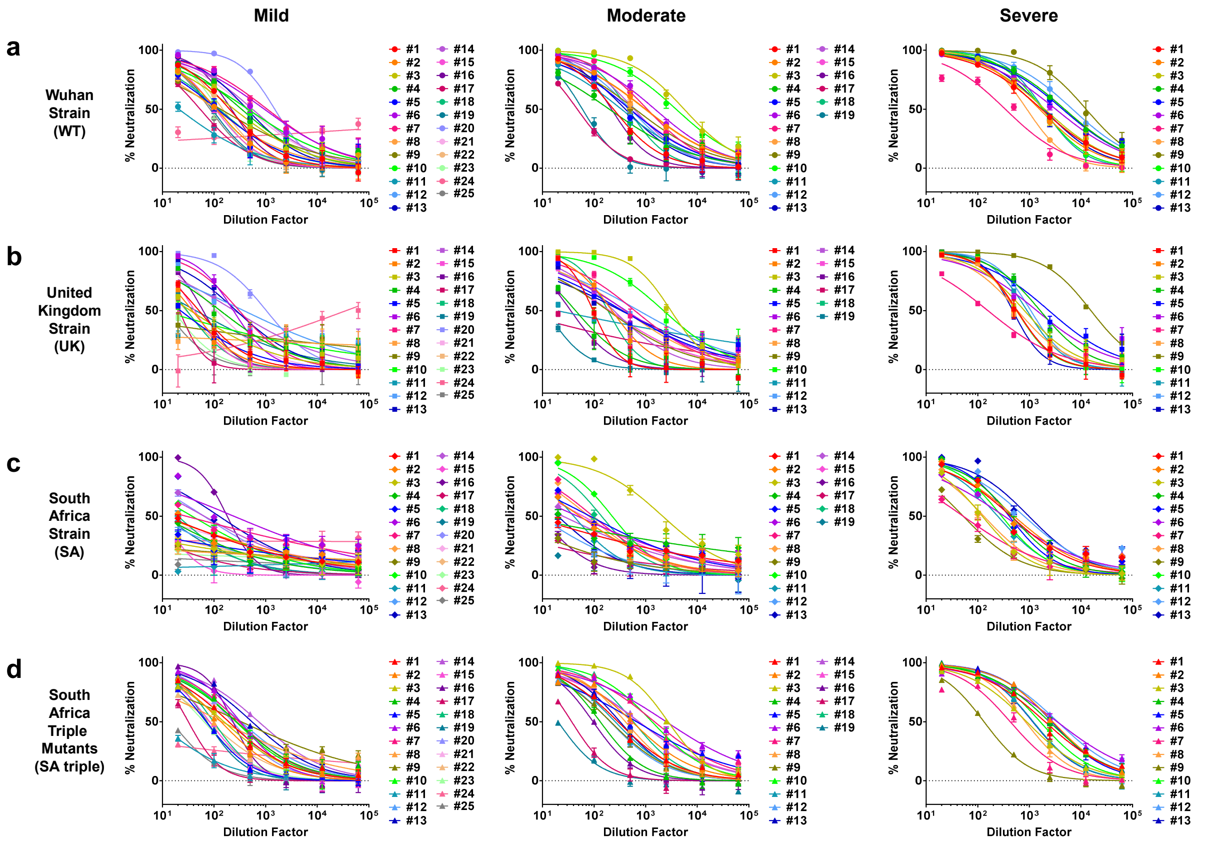

Supplementary Figure 1: Pseudovirus neutralization curves by 57 COVID-19 convalescent plasma samples. Plasma samples of Mild (n=25), Moderate (n=19), and Severe (n=13) COVID-19 patients at median 31 days post-illness onset (pio) were incubated with pseudovirus expressing SARS-CoV-2 spike (S) glycoprotein 1 hour prior to infection of CHO-ACE2 cells for 48 hours. Infection levels were determined by luciferase assay, and percentage of neutralization is presented. Dose-response titration curves against (**a**) wildtype Wuhan strain (WT, circles), (**b**) UK strain (B.1.1.7, squares), (**c**) SA strain (B.1.351, diamonds) and (**d**) SA triple mutant (K417N/E484K/N501Y, triangles) at 1:20 to 1:62500 dilutions. Lines represent non-linear regression fit and data are shown as mean ± SD of two to four independent experiments.

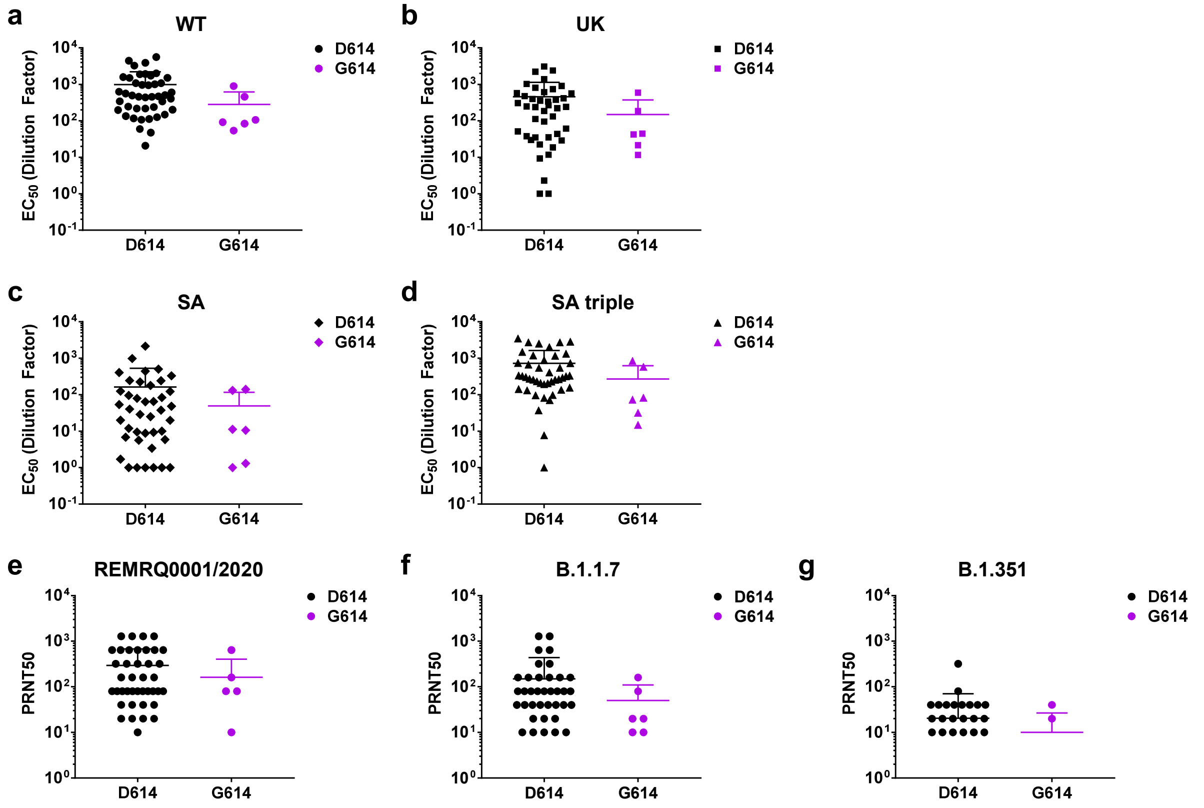

Supplementary Figure 2: Effect of initial infection status on antibody neutralization against different pseudovirus strains. Dot plots of EC_{50} values of neutralization against (**a**) WT (circles), (**b**) UK (squares), (**c**) SA (diamonds) and (**d**) SA triple (triangles) pseudoviruses from COVID-19 patients presented in two different groups of initial infection status of D614 strain (n=44, black), and G614 strain (n=6, purple). Dot plots of PRNT50 values of live virus neutralization against (**e**) RMERQ0001/2020, (**f**) B.1.1.7 isolate, and (**g**) B.1.351 isolate from COVID-19 patients presented in two different groups of initial infection status of D614 strain (n=44, black), and G614 strain (n=6, purple). Since results are plotted on a logarithmic scale and plasma samples that did not neutralize the live virus with assigned PRNT50 values at “0” are not plotted in (**e-g**). Statistical analysis was carried out to compare D614 and G614 groups of patients using Kruskal-Wallis tests followed by post hoc Dunn’s multiple comparisons tests and there is no significant difference between D614 and G614 group of patients against all different pseudovirus or live virus strains.

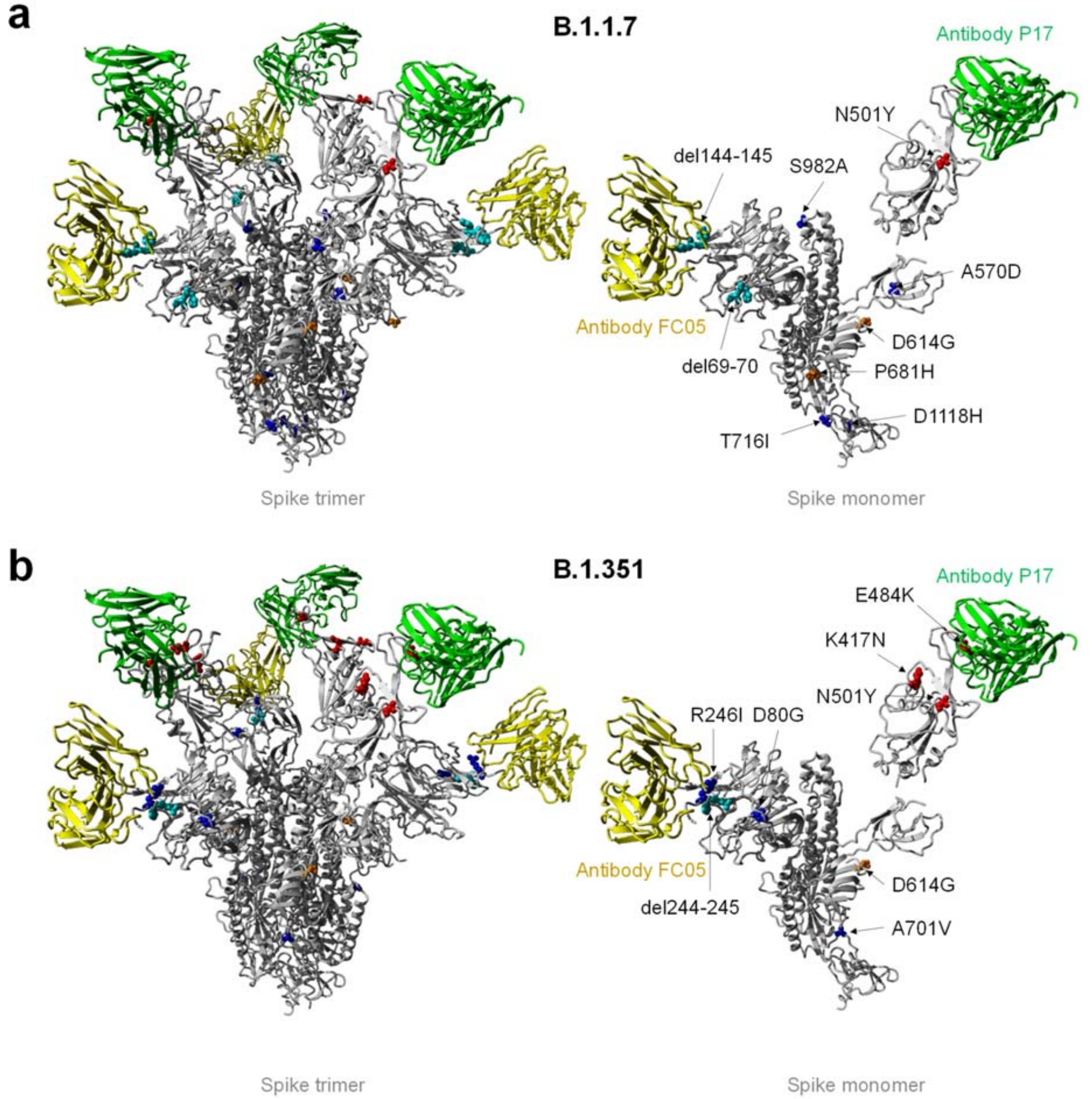

Supplementary Figure 3: Spike glycoprotein from PDB:7CWU in complex with 2 antibodies. Spike glycoprotein in gray, 2 antibodies in green (P17) and yellow (FC05), and amino acid changes corresponding to B.1.1.7 (**a**) or B.1.351 (**b**) are highlighted in colored balls with position labels. Left panel: spike trimer, right panel: spike monomer. Visualization with Yasara.

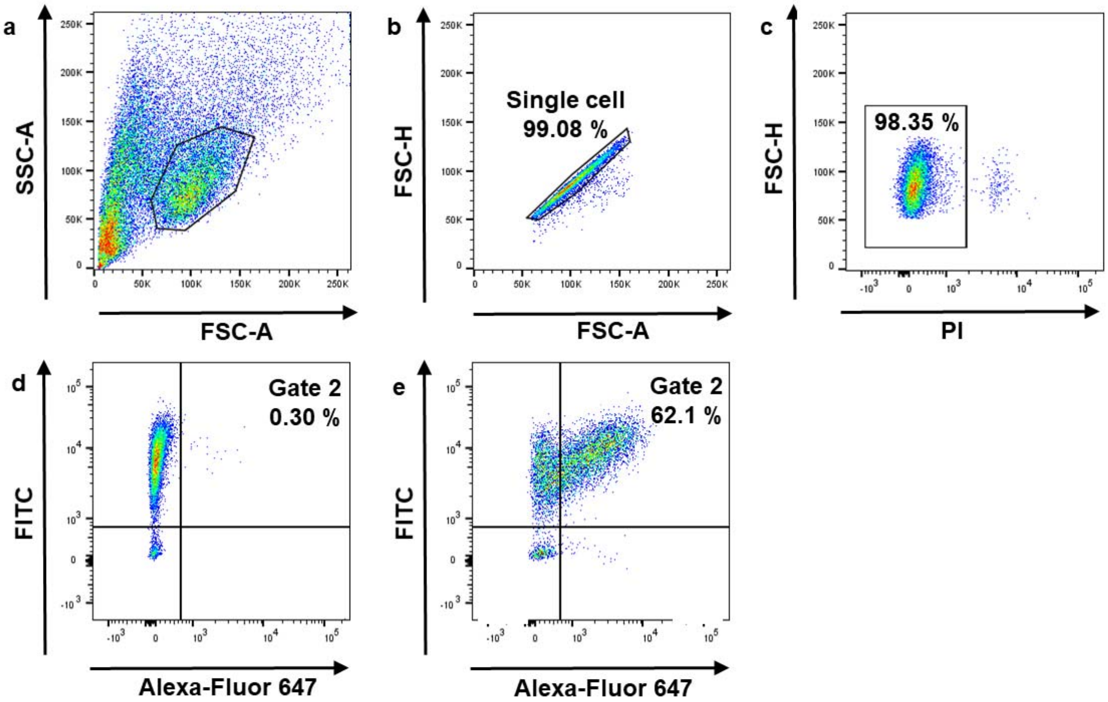

Supplementary Figure 4: FACS plot analysis. Cells were gated on: (**a**) FSC-A/SSC-A to exclude cell debris, (**b**) FSC-A/FSC-H to select for single cells, (**c**) FSC-A/PI to select for live cells (PI-negative population), (**d**, **e**) FITC/Alexa Fluor 647. Binding is determined by the percentage of GFP-positive S protein-expressing cells that are bound by specific antibody, indicated by the events that are Alexa Fluor 647- and FITC-positive (Gate 2). (**d**) healthy control plasma, 1:100 diluted; (**e**) COVID-19 patient plasma, 1:100 diluted.

SUPPLEMENTARY TABLES

Supplementary Table 1: Pseudovirus neutralization EC_{50} values of for 57 COVID-19 convalescent plasma samples.

Patient	WT	UK	SA	SA triple
**Mild (#1)**	200.6	51.1	11.9	193.1
**Mild (#2)**	112.0	37.9	9.5	136.4
**Mild (#3)**	245.7	35.0	1.0*	155.9
**Mild (#4)**	342.1	132.1	10.0	273.0
**Mild (#5)**	116.5	22.5	1.0*	70.6
**Mild (#6)**	948.5	237.7	241.8	323.7
**Mild (#7)**	1077.0	306.9	28.8	304.5
**Mild (#8)**	106.7	1.0*	1.0*	142.7
**Mild (#9)**	134.7	1.0*	1.0*	337.6
**Mild (#10)**	458.8	9.3	37.6	266.2
**Mild (#11)**	20.8	N.D.	N.D.	7.7
**Mild (#12)**	454.5	377.8	6.8	331.3
**Mild (#13)**	439.3	269.0	125.3	544.6
**Mild (#14)**	558.7	247.8	64.7	734.6
**Mild (#15)**	106.3	42.1	10.6	82.3
**Mild (#16)**	217.5	61.2	179.9	233.1
**Mild (#17)**	53.9	21.4	1.3	31.8
**Mild (#18)**	125.5	29.8	20.0	97.7
**Mild (#19)**	91.6	44.4	11.2	73.0
**Mild (#20)**	1504.0	1032.0	63.8	228.2
**Mild (#21)**	468.7	112.2	5.9	256.4
**Mild (#22)**	148.2	29.0	1.0*	81.3
**Mild (#23)**	122.6	16.3	1.0*	64.0
**Mild (#24)**	N.D.	N.D.	1.0*	1.0*
**Mild (#25)**	83.7	11.6	1.0*	14.8
**Moderate (#1)**	238.4	95.7	5.6	291.9
**Moderate (#2)**	1007.0	221.8	53.4	545.8
**Moderate (#3)**	5586.0	3086.0	2136.0	2732.0
**Moderate (#4)**	197.7	43.8	3.4	131.8
**Moderate (#5)**	495.7	356.4	83.5	670.4
**Moderate (#6)**	1514.0	414.6	48.1	2509.0
**Moderate (#7)**	1076.0	614.3	95.3	1485.0
**Moderate (#8)**	970.5	396.7	79.2	405.4
**Moderate (#9)**	628.1	186.7	1.7	209.6
**Moderate (#10)**	3895.0	2220.0	224.6	1327.0
**Moderate (#11)**	340.2	18.7	40.3	257.0
**Moderate (#12)**	508.1	737.9	8.8	205.6
**Moderate (#13)**	405.1	338.2	19.9	318.2
**Moderate (#14)**	599.1	241.9	25.0	739.0
**Moderate (#15)**	510.4	200.8	61.5	736.1
**Moderate (#16)**	217.9	35.3	9.3	95.9
**Moderate (#17)**	47.4	2.3	1.0*	36.9
**Moderate (#18)**	458.2	186.0	139.8	580.2
**Moderate (#19)**	59.5	11.8	N.D.	20.0
**Severe (#1)**	1737.0	579.7	441.3	2021.0
**Severe (#2)**	1582.0	564.6	504.5	2850.0
**Severe (#3)**	2006.0	804.4	122.6	888.9
**Severe (#4)**	4839.0	1626.0	459.4	2449.0
**Severe (#5)**	4439.0	2401.0	406.0	2730.0
**Severe (#6)**	3257.0	1393.0	328.6	3471.0
**Severe (#7)**	343.2	157.3	52.2	427.1
**Severe (#8)**	897.9	590.1	131.3	840.0
**Severe (#9)**	12614.0	15629.0	52.4	132.3
**Severe (#10)**	1822.0	568.2	291.1	1623.0
**Severe (#11)**	1896.0	905.1	244.0	1194.0
**Severe (#12)**	6882.0	1067.0	793.5	3539.0
**Severe (#13)**	1918.0	477.4	978.9	1166.0

* Use 1.0 if calculated $EC_{50} < 1$; N.D., not determined.

Supplementary Table 2: Live virus neutralization PRNT50 values of for 57 COVID-19 convalescent plasma samples.

Patient	REMRQ0001/2020	B.1.1.7	B.1.351
**Mild (#1)**	160	160	0
**Mild (#2)**	20	160	10
**Mild (#3)**	80	20	0
**Mild (#4)**	20	10	20
**Mild (#5)**	0	0	0
**Mild (#6)**	80	80	40
**Mild (#7)**	40	160	10
**Mild (#8)**	0	0	0
**Mild (#9)**	80	0	0
**Mild (#10)**	640	40	0
**Mild (#11)**	0	0	0
**Mild (#12)**	80	10	0
**Mild (#13)**	320	40	20
**Mild (#14)**	80	40	10
**Mild (#15)**	80	10	0
**Mild (#16)**	80	160	20
**Mild (#17)**	10	10	0
**Mild (#18)**	160	10	0
**Mild (#19)**	160	20	0
**Mild (#20)**	640	640	0
**Mild (#21)**	40	80	0
**Mild (#22)**	20	80	0
**Mild (#23)**	20	20	0
**Mild (#24)**	0	0	0
**Mild (#25)**	0	20	0
**Moderate (#1)**	80	160	0
**Moderate (#2)**	160	80	20
**Moderate (#3)**	640	1280	320
**Moderate (#4)**	40	40	0
**Moderate (#5)**	80	40	10
**Moderate (#6)**	40	40	0
**Moderate (#7)**	80	80	20
**Moderate (#8)**	1280	0	20
**Moderate (#9)**	640	80	0
**Moderate (#10)**	1280	80	40
**Moderate (#11)**	80	10	0
**Moderate (#12)**	320	20	0
**Moderate (#13)**	80	20	10
**Moderate (#14)**	320	40	40
**Moderate (#15)**	80	20	80
**Moderate (#16)**	640	40	0
**Moderate (#17)**	20	0	0
**Moderate (#18)**	80	80	40
**Moderate (#19)**	10	10	0
**Severe (#1)**	1280	1280	40
**Severe (#2)**	640	640	40
**Severe (#3)**	160	320	10
**Severe (#4)**	320	320	80
**Severe (#5)**	1280	80	40
**Severe (#6)**	640	320	40
**Severe (#7)**	320	40	0
**Severe (#8)**	640	160	20
**Severe (#9)**	5120	5120	0
**Severe (#10)**	320	320	40
**Severe (#11)**	320	80	40
**Severe (#12)**	640	320	160
**Severe (#13)**	320	160	80

Use value “0” if the plasma samples failed to neutralize 50% of live virus at the lowest plasma dilution.

Supplementary Table 3: Primers used in this study.

Primer Name	Primer Sequences
FL1- del69-70-F	GACATGGTTTCACGCGATCTCAGGCACTAACGGGACGA
FL1-del144-145-F	CGACCCGTTCCTGGGCGTGCATAAGAATAATAAGTCAT
FL1-K417N-F	CGGACAGACCGGGAAcATCGCCGACTACAAC
FL1-E484K-F	CTACCCCATGCAACGGAGTGaAAGGGTTCAACTGCTATTTC
FL1-N501Y-F	CTACGGGTTTCAGCCAACCtACGGCGTCGGATATCAGCC
FL1-D614G-F	AAGTAGCCGTCTTGTACCAAGgCGTCAATTGTACCGAGGTGCC
FL1-A570D-F	TTCGGTCGCGATATCGaCGATACAACAGACGCCGTTC
FL1-P681H-F	CAAACCCAAACAAACTCACaCAGGCGGGCTAGGTCTGTT
FL1-T716I-F	ACAATTCCATAGCTATTCCTAtCAACTTCACAATCTCCGTAACT
FL1-S982A-F	GCGTGTTGAACGACATATTGgcCAGGCTCGATAAGGTAGAAG
FL1-D1118H-F	AGCCCCAGATTATCACCACTcATAATACCTTCGTCAGCGGA
FL1-D80A-F	GGACGAAACGATTCGcCAATCCGGTGCTCCC
FL1-R246I-F	CACTGTTGGCCCTGCACatcTCTTATCTCACCCCGGG
FL1-A701V-F	CCATGTCCCTGGGCGtTGAGAACAGTGTGGC
FL1-del242-245-F	GGTTCCAGACACTGatcTCTTATCTCACCCC
XbaSPFor	GCTCTAGAGCCACCATGTTCGTCTTCCTGGTGC
BamSPRev	CGGGATCCttaACAACAAGAGCCGCAGG
SPF1	ATGTTCGTCTTCCTGGTG
SPF2	CCTGTGTTTCACAAACGTGT
SPF3	GATACAACAGACGCCGTTC
SPF4	CAGGCCTTGAATACCCTCG
IRESrev	CATATAGACAAACGCACACC
