# Supplementary material for: Resistance of SARS-CoV-2 variants to neutralization by convalescent plasma from early COVID-19 outbreak in Singapore
Source: NPJ Vaccines. 2021 Oct 25;6:125. doi: 10.1038/s41541-021-00389-2 (PMC8546091; doi:10.1038/s41541-021-00389-2)
Supplement: Supplementary file 2 — Reporting Summary [file 41541_2021_389_MOESM2_ESM.pdf]

nature portfolio

Corresponding author(s): Cheng-I Wang; Laurent Renia

Last updated by author(s): Sep 9, 2021

Reporting Summary

Nature Portfolio wishes to improve the reproducibility of the work that we publish. This form provides structure for consistency and transparency in reporting. For further information on Nature Portfolio policies, see our Editorial Policies and the Editorial Policy Checklist.

Statistics

For all statistical analyses, confirm that the following items are present in the figure legend, table legend, main text, or Methods section.

n/a	Confirmed	
☐	☒	The exact sample size (n) for each experimental group/condition, given as a discrete number and unit of measurement
☐	☒	A statement on whether measurements were taken from distinct samples or whether the same sample was measured repeatedly
☐	☒	The statistical test(s) used AND whether they are one- or two-sided<br>*Only common tests should be described solely by name; describe more complex techniques in the Methods section.*
☐	☒	A description of all covariates tested
☐	☒	A description of any assumptions or corrections, such as tests of normality and adjustment for multiple comparisons
☐	☒	A full description of the statistical parameters including central tendency (e.g. means) or other basic estimates (e.g. regression coefficient) AND variation (e.g. standard deviation) or associated estimates of uncertainty (e.g. confidence intervals)
☒	☐	For null hypothesis testing, the test statistic (e.g. F, t, r) with confidence intervals, effect sizes, degrees of freedom and P value noted<br>*Give P values as exact values whenever suitable.*
☒	☐	For Bayesian analysis, information on the choice of priors and Markov chain Monte Carlo settings
☒	☐	For hierarchical and complex designs, identification of the appropriate level for tests and full reporting of outcomes
☐	☒	Estimates of effect sizes (e.g. Cohen's d, Pearson's r), indicating how they were calculated

Our web collection on statistics for biologists contains articles on many of the points above.

Software and code

Policy information about availability of computer code

Data collection	n/a
Data analysis	n/a

For manuscripts utilizing custom algorithms or software that are central to the research but not yet described in published literature, software must be made available to editors and reviewers. We strongly encourage code deposition in a community repository (e.g. GitHub). See the Nature Portfolio guidelines for submitting code & software for further information.

Data

Policy information about availability of data

All manuscripts must include a data availability statement. This statement should provide the following information, where applicable:

- Accession codes, unique identifiers, or web links for publicly available datasets
- A description of any restrictions on data availability
- For clinical datasets or third party data, please ensure that the statement adheres to our policy

The data generated and analyzed during the current study are available from the corresponding author upon reasonable request.

Field-specific reporting

Please select the one below that is the best fit for your research. If you are not sure, read the appropriate sections before making your selection.

☒ Life sciences ☐ Behavioural & social sciences ☐ Ecological, evolutionary & environmental sciences

For a reference copy of the document with all sections, see nature.com/documents/nr-reporting-summary-flat.pdf

Life sciences study design

All studies must disclose on these points even when the disclosure is negative.

Sample size	No sample size calculation was performed.
Data exclusions	For pseudovirus neutralization assays, the EC50 values for some samples cannot be determined due to weak/no neutralization activity. These include 1 sample for WT strain, 2 samples for UK strain, and 2 samples for SA strain respectively (see Supplementary Table 1) . Hence, in the following paired analysis of EC50 values (Fig. 2e-2f), heat map presentation of Log10 values of fold increase or decrease of EC50 values (Fig.2g) as well as the correlation analysis between pseudovirus EC50 values and live virus PRNT50 values (Fig.4g), those data points associated with these samples were excluded and not shown in the figures. In addition, for live virus neutralization assay, since results are plotted on a logarithmic scale and plasma samples that did not neutralize the live virus with assigned PRNT50 values at "0" are not plotted in Fig.4a-4d. We have clearly made the statement of data exclusions in the figure legends.
Replication	For SFB assay, data are shown as mean ± SD of two independent experiments. For pseudovirus neutralization assay, data are shown as mean ± SD of two to four independent experiments. N numbers for each datasets are reported in the figure legends.
Randomization	n/a
Blinding	n/a

Reporting for specific materials, systems and methods

We require information from authors about some types of materials, experimental systems and methods used in many studies. Here, indicate whether each material, system or method listed is relevant to your study. If you are not sure if a list item applies to your research, read the appropriate section before selecting a response.

Materials & experimental systems

n/a	Involved in the study	
☐	☒	Antibodies
☐	☒	Eukaryotic cell lines
☒	☐	Palaeontology and archaeology
☒	☐	Animals and other organisms
☐	☒	Human research participants
☒	☐	Clinical data
☒	☐	Dual use research of concern

Methods

n/a	Involved in the study	
☒	☐	ChIP-seq
☐	☒	Flow cytometry
☒	☐	MRI-based neuroimaging

Antibodies

Antibodies used	Alexa Fluor 647-conjugated anti-human IgM (Thermo Fisher Scientific, Cat#A21249, RRID: AB_2535817); Alexa Fluor 647-conjugated anti-human IgG (Thermo Fisher Scientific, Cat#A21445, RRID: AB_2535862)
Validation	No commercial primary antibodies were used in this study.

Eukaryotic cell lines

Policy information about cell lines

Cell line source(s)	HEK293T from ATCC; Vero C1008 from Public Health England (PHE); CHO-ACE2 cell line is a gift from Prof. Yee-Joo Tan (NUS & IMCB)
Authentication	Cell lines were not authenticated. We obtained them either from commercial sources or another lab.
Mycoplasma contamination	All cell lines used in this study have been tested to be mycoplasma negative.

Commonly misidentified lines (See ICLAC register)	No commonly misidentified cell lines were used in this study.

Human research participants

Policy information about studies involving human research participants

Population characteristics	In this study, we used the same cohort of 57 COVID-19 patients as we used in our previous study (doi: 10.1002/cti2.1241). The covariant-relevant population characteristics of the participants (age, gender, genotypic information etc) can be found in Table 1 of that publication.
Recruitment	Fifty-seven patients who tested PCR-positive for SARS-CoV-2 in nasopharyngeal swabs in Singapore were recruited into the study from January to March 2020. Patients were categorised into three groups based on clinical severity during hospitalisation: mild (no pneumonia on chest radiographs (CXR), n = 25), moderate (pneumonia on CXR without hypoxia, n = 19) and severe (pneumonia on CXR with hypoxia (desaturation to ≤ 94%), n = 13). There are no self-selection biases that may be likely to impact results.
Ethics oversight	The design and protocols of this study for convalescent COVID-19 patient cohorts have been approved by National Healthcare Group (NHG) Domain Specific Review Board (DSRB) under study number IRB#2012/00917, and performed following the ethical guidelines. Written informed consent was obtained from participants in accordance with the tenets of the Declaration of Helsinki. All samples received were collected under Singapore Infectious Diseases Act, which allows epidemiological studies and use of data for analysis to control disease outbreaks.

Note that full information on the approval of the study protocol must also be provided in the manuscript.

Flow Cytometry

Plots

Confirm that:

- [x] The axis labels state the marker and fluorochrome used (e.g. CD4-FITC).
- [x] The axis scales are clearly visible. Include numbers along axes only for bottom left plot of group (a 'group' is an analysis of identical markers).
- [x] All plots are contour plots with outliers or pseudocolor plots.
- [x] A numerical value for number of cells or percentage (with statistics) is provided.

Methodology

Sample preparation	Human cell line HEK293T was used in this study to generate S protein-expressing cell line by lentiviral transduction. Then the S protein-expressing cell lines were dissociated from the culture flask by trypsinization and were seeded in 96 well V-bottom plates for incubation with diluted patient plasma.
Instrument	BD LSRII 4-lazer analyzer
Software	FlowJo (Tree Star)
Cell population abundance	95-100%
Gating strategy	Cells were gated on: (1) FSC-A/SSC-A to exclude cell debris, (2) FSC-A/FSC-H to select for single cells, (3) FSC-A/PI to select for live cells (PI-negative population), (4) FITC/Alexa Fluor 647.

- [x] Tick this box to confirm that a figure exemplifying the gating strategy is provided in the Supplementary Information.
